# Cystic Lymphangioma as a Cause of Massive Abdominal Hemorrhage

**DOI:** 10.1155/2011/492564

**Published:** 2011-07-03

**Authors:** E. Consuegra Llapur, J. M. López Alvarez, O. Pérez Quevedo, M. Valerón Lemaur, S. Pavlovic Nesic, X. García Urgellés

**Affiliations:** ^1^Pediatric Intensive Care Unit, Hospital Universitario Materno-Infantil de Canarias, 35010 Las Palmas de GC, Spain; ^2^Pediatric Emergency Service, Hospital Universitario Materno-Infantil de Canarias, 35010 Las Palmas de GC, Spain; ^3^Pediatric Surgery Unit, Hospital Universitario Materno-Infantil de Canarias, 35010 Las Palmas de GC, Spain

## Abstract

We report the case of two-year-old girl with hypovolemic shock caused by bleeding from an abdominal cystic lymphangioma. The whole blood was contained within a large omental bag that could be completely removed. There were no associated anomalies. The child progressed satisfactorily.

## 1. Introduction

Abdominal cystic lymphangioma is not a common disorder during childhood. It usually presents with abdominal pain or acute abdomen but also presents as an asymptomatic mass.

## 2. Clinical Case

**Figure 1:**
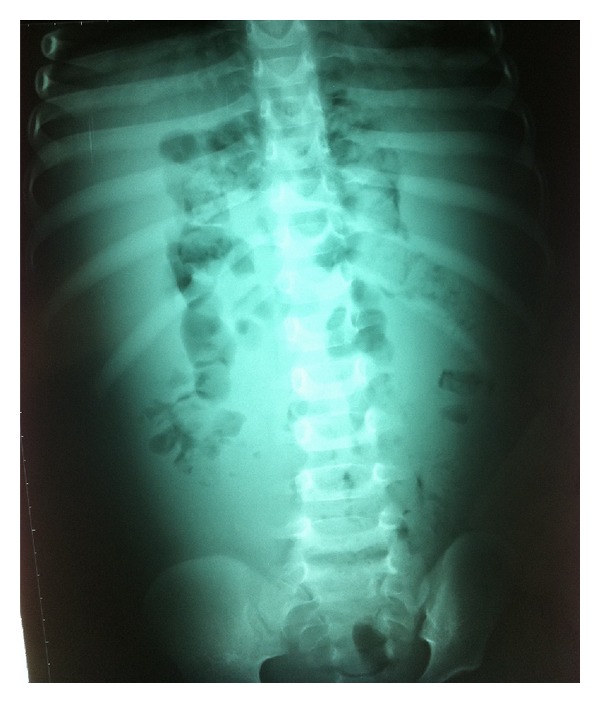
Medial displacement of the colic frame.

**Figure 2:**
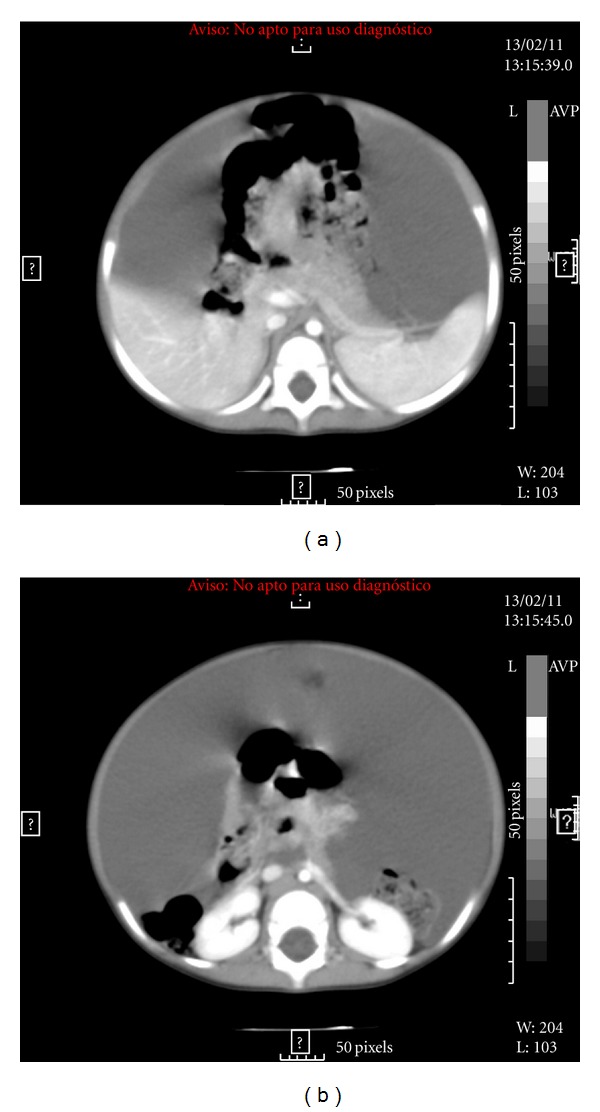
Bleeding tension, displacing abdominal organs.

**Figure 3:**
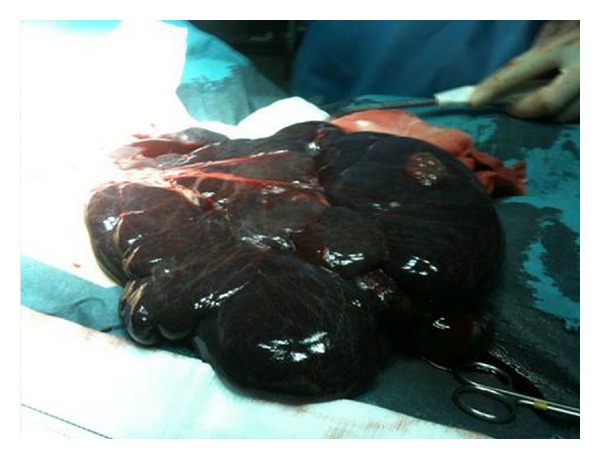
Membranous bag containing blood.

Two-year-old girl, whose father died of an aortic aneurysm in Marfan's disease, showed progressive enlargement of the abdomen, as well as pallor and fatigue of at least two weeks later. She was brought to the emergency department with weakness, apathy, and pallor. AP abdominal radiograph (Figure 1) shows medial displacement of the colic frame and presence of gas in distal large intestine as radiopacity of the abdomen. CBC: Hb of 3.4 g/dL. Abdominal US: loss of normal positioning of viscera and fluid image that fills the intraperitoneal space. Abdominal CT findings show the presence of tension liquid that displaces the organs, but they retain their normal appearance (Figure 2), because the presence of clinical signs of hypovolemic shock is performed volume expansion with saline and packed red blood cells by central (right subclavian). Then it is taken to OR; exploratory laparotomy revealed the presence of a membranous bag containing about 2 liters of blood (Figure 3). The full bag was removed. It was consisted of the omental wall. A blood vessel bleeding was found which happens to be a branch of splenic artery. Ligation was performed. She was transfused with 2 units of packed red blood cells and transferred to the intensive care unit sedated and with mechanical ventilation. She was extubated 3 hours after arrival of surgery, after which she progressed satisfactorily. Post-transfusion Hb: 14.2 gm. The next day she was transferred to pediatric ward and finally discharged on the fifth day without complications.

## 3. Discussion

Lymphangiomas are benign tumors of the lymphatic vessels usually located in subcutaneous tissue of head, neck and armpit, and more rarely in abdomen [1–5], being 5% of reported cases with this location [6].

The pathological examination of the masses located in abdomen usually shows thin-walled cavities of different sizes and may contain lymph, pus when infected, or blood as a result of bleeding [1]. Our case has special features that merit consideration, first its debut through bleeding such that causes hypovolemic shock [7, 8] and second the preservation of the integrity of the wall of the tumor despite the heightened tension caused by the blood contained, so that allowed full removal.

Cystic Lymphangioma should be considered as a possible cause of massive abdominal hemorrhage.
